# Fabrication and Impact of Fouling-Reducing Temperature-Responsive POEGMA Coatings with Embedded CaCO_3_ Nanoparticles on Different Cell Lines

**DOI:** 10.3390/ma14061417

**Published:** 2021-03-15

**Authors:** Ostap Lishchynskyi, Yurij Stetsyshyn, Joanna Raczkowska, Kamil Awsiuk, Barbara Orzechowska, Anatolii Abalymov, Andre G. Skirtach, Andrzej Bernasik, Svyatoslav Nastyshyn, Andrzej Budkowski

**Affiliations:** 1 Department of Organic Chemistry, Lviv Polytechnic National University, St. George’s Square 2, 79-013 Lviv, Ukraine; oslishchynskyi@gmail.com; 2 Smoluchowski Institute of Physics, Jagiellonian University, Łojasiewicza 11, 30-348 Kraków, Poland; kamil.awsiuk@uj.edu.pl (K.A.); svyatoslav.nastyshyn@doctoral.uj.edu.pl (S.N.); andrzej.budkowski@uj.edu.pl (A.B.); 3 Institute of Nuclear Physics Polish Academy of Sciences, Radzikowskiego 152, 31-342 Kraków, Poland; barbara.orzechowska@ifj.edu.pl; 4 Department of Biotechnology, Ghent University, Coupure Links 653, 9000 Ghent, Belgium; anatolii.abalymov@ugent.be (A.A.); andre.skirtach@ugent.be (A.G.S.); 5 Faculty of Physics and Applied Computer Science, AGH—University of Science and Technology, Al. Mickiewicza 30, 30-049 Kraków, Poland; bernasik@agh.edu.pl

**Keywords:** grafted coatings, nanoparticles, cells, poly(di(ethylene glycol)methyl ether methacrylate), cell adhesion, HaCaT, WM35, MC3T3-e1

## Abstract

In the present work, we have successfully prepared and characterized novel nanocomposite material exhibiting temperature-dependent surface wettability changes, based on grafted brush coatings of non-fouling poly(di(ethylene glycol)methyl ether methacrylate) (POEGMA) with the embedded CaCO_3_ nanoparticles. Grafted polymer brushes attached to the glass surface were prepared in a three-step process using atom transfer radical polymerization (ATRP). Subsequently, uniform CaCO_3_ nanoparticles (NPs) embedded in POEGMA-grafted brush coatings were synthesized using biomineralized precipitation from solutions of CaCl_2_ and Na_2_CO_3_. An impact of the low concentration of the embedded CaCO_3_ NPs on cell adhesion and growth depends strongly on the type of studied cell line: keratinocytes (HaCaT), melanoma (WM35) and osteoblastic (MC3T3-e1). Based on the temperature-responsive properties of grafted brush coatings and CaCO_3_ NPs acting as biologically active substrate, we hope that our research will lead to a new platform for tissue engineering with modified growth of the cells due to the release of biologically active substances from CaCO_3_ NPs and the ability to detach the cells in a controlled manner using temperature-induced changes of the brush.

## 1. Introduction

Modern biomaterials allow a deeper understanding of the interaction within biological systems at both the cellular and molecular levels. The ultimate goal of such studies is to create materials and products, which are better suitable for diverse applications in biomedicine. In the past few years, such materials were created on the basis of the nanocoatings [1,2,3], grafted polymer brushes [4,5,6,7], nanotubes [8,9,10,11], hydrogels [12,13], organic and inorganic nanoparticles [14,15], and others. To improve biological action of polymeric materials, they are often modified in different manners. The functionalization of polymeric matrices can be performed by various components, for example: drugs and biomolecules absorbed on the scaffold surface and/or loaded to the scaffold interior; inorganic micro- and nanoparticles embedded, absorbed or synthesized on scaffold surface; coatings on the scaffold surface.

It is well known that some of the most important advantages of nanoparticles are their size, their surface area, their capability to transfer and protect biomolecules from degradations, as well as “preservation” of their properties and a controlled release over time, their locality of action and specificity of interactions with biological structures [16]. The main requirements for nanoparticles for their use in medicine are their low toxicity, high biocompatibility, their capability to degrade or to be excreted naturally. Nanoparticles may be divided into two main types, based on their nature, i.e., organic [17,18] and inorganic [19,20] ones. Inorganic particles and structures include, for example, nanosized particles of calcium or strontium carbonate [21,22,23,24], gold and silver [25,26,27] and iron oxide [28], as well as quantum dots and carbon nanotubes [29,30], and others. In turn, hybrid nanomaterials, i.e., materials composed of both inorganic and organic components, provide the ability to tune the properties of the hybrid material systematically and are therefore very promising for imaging and therapeutic applications [31]. Hybrid materials based on a combination of organic (including chitosan) and inorganic hydroxyapatite components have been used to repair bones, which consist of 60–65% of hydroxyapatite, collagen, chondroitin sulfate, etc. [32]. Hybrid nanosystems are also used in effective cancer treatment approaches to deliver the important anti-cancer therapeutic agents [33]. Calcium carbonates represent an attractive type of inorganic particles due to their biocompatibility, biodegradability and accessibility [34]. As such, a high number of applications have been demonstrated including their application for removal of toxic heavy metal ions from aqueous solutions [35]. Moreover, injectable, self-gelling hydrogel–microparticle composites and hybrid scaffolds for bone regeneration [36] and capabilities of carrying biologically active molecules [37] have been developed in recent years. These composites include calcium and magnesium carbonates, alpha-tricalcium phosphate, or their combinations [36,37,38]. All such composites are highly cytocompatible. In other reports, novel composite scaffolds based on polymeric polycaprolactone or poly(3-hydroxybutyrate) fibers coated with porous calcium carbonate structures (PCL/CaCO_3_) were applied for tissue engineering, and their drug delivery capacity was demonstrated [38]. In addition, composite materials containing hydroxyapatite or calcium phosphate nanoparticles play an important role in various biomedical applications, mainly due to the excellent biocompatibility of hydroxyapatite nanoparticles (HA NPs) [39]. The cell culture experiments, performed by the group of S. Wei, showed improved cytophilicity of the nanophase mineral as compared to conventional hydroxyapatite [40,41]. Moreover, calcium phosphate-based systems are osteoconductive, osteoinductive, and the majority are considered bioresorbable, which is essential for new, effective therapies for bone regeneration [42].

On the other hand, great attention is devoted to the non-fouling polymer materials [43], especially grafted polymer brushes, which show surface resistance to nonspecific protein adsorption, cell/bacterial adhesion, and biofilm formation that is critical for the development and performance of biomedical and analytical devices. Non-fouling properties are strongly correlated with the ability to form a hydration layer near the polymer surfaces, resulting in a physical and energetic barrier that prevents protein adsorption and cell adhesion on the surfaces. The low-protein adsorption on the surface modified with polymer brushes of methoxy- and hydroxy-capped oligoethylene glycol methacrylate, 2-hydroxyethyl methacrylate and carboxybetaine acrylamide was also reported [44,45,46]. In addition, the behavior of cells on non-fouling poly(di(ethylene glycol)methyl ether methacrylate) (POEGMA)-modified surfaces with different topographies was investigated [47], where it was found that cells adherent to topographical surfaces were more firmly attached compared to those on smooth surfaces. In turn, a study on the anti-fouling properties of three types of poly(oligo(ethylene glycol) methyl ether methacrylate) (OEGMA)-grafted brush coatings was conducted [48] revealing that cell-fouling resistance strongly depends on ethylene glycol side chain length. This issue was studied for oligo(ethylene glycol) methyl ether methacrylate monomers containing side chains of 4, 9, and 23 ethylene glycol units. In our previous works [49], we fabricated anti-fouling, temperature-responsive grafted polymer brush coatings with embedded silver nanoparticles for thermo-switchable biological activity and demonstrated that the impact of AgNPs in grafted brush coatings is highly cell dependent. In contrast to scaffold-based tissue engineering, a tissue engineering methodology where cells have been cultured on an intelligent cell culture surface modified by temperature-responsive grafted polymer brushes have numerous advantages [50]. Using this methodology allows us to omit a pathological state of fibrosis due to the low cell density of constructed tissue, necrosis caused by lack of microcapillaries and strong inflammatory responses due to the biodegradation of scaffolds [50]. N-isopropylacrylamide and oligo(ethylene glycol) methacrylates are the most widely used monomers for construction of temperature-responsive polymer brush coatings for tissue engineering [51].

Motivated by the extensive progress in research on materials containing CaCO_3_ for biomedical applications, in the present work, we have prepared and characterized temperature-responsive grafted brush coatings of poly(di(ethylene glycol)methyl ether methacrylate) (POEGMA). The influence of the low concentration of the embedded CaCO_3_ nanoparticles (CaCO_3_ NPs), as well as their impact on adhesion and growth of the different cellular types, was investigated here. The composition, thickness, morphology and wettability of the resulting coatings were analyzed using time of flight secondary ion mass spectroscopy (ToF-SIMS), X-ray photoelectron spectroscopy (XPS), ellipsometry, scanning electronic microscopy (SEM), atomic force microscopy (AFM) and contact angle (CA) measurements, respectively. In turn, an impact of the low concentration of the embedded CaCO_3_ NPs on cell adhesion and growth was studied in details for three cell lines: keratinocytes from histologically normal skin (HaCaT), WM35 cell line from the primary melanoma site of the patient’s skin diagnosed with radial growth phase (RGP) melanoma and osteoblastics (MC3T3-e1). Cells were cultured on glass substrates modified with grafted POEGMA brush coatings and POEGMA brush coatings containing low concentrations of the embedded CaCO_3_ nanoparticles.

## 2. Materials and Methods

### 2.1. Materials

(3-aminopropyl)triethoxysilane (APTES), 2-bromoisobutyryl bromide (BIBB), triethylamine (Et_3_N), di(ethylene glycol) methyl ether methacrylate) (DEGMEM), sodium L-ascorbate, CuBr_2_, 2,2′-dipyridyl (bpy), CaCl_2_, Na_2_CO_3_ and solvents were purchased from Sigma-Aldrich (Darmstadt, Germany).

### 2.2. Fabrication of Coatings with CaCO_3_ NPs

#### 2.2.1. Preparation of Coatings: Modification of Glass Surfaces with ATRP Initiator

Glass plates (20 mm × 20 mm) were placed in a vacuum oven with a vial containing 10 drops of APTES. The chamber was pumped down to < 1 mbar, isolated from the pump and left under a vacuum for 30 min. Then, substrates were annealed at 110 °C in air at atmospheric pressure for 30 min.

After annealing, substrates can be reacted directly with 2-bromoisobutyryl bromide. For this, 10 mL anhydrous tetrahydrofuran was mixed with 2-bromoisobutyryl bromide (0.26 mL, 2.10 mmol) and anhydrous triethylamine (0.30 mL, 2.10 mmol) and the mixture was added to the amino functionalized substrate.

#### 2.2.2. Polymerization of POEGMA Brushes (Surface-Initiated Activators ReGenerated by Electron Transfer Atom Transfer Radical Polymerization (SI-ARGET ATRP))

**Scheme 1:**
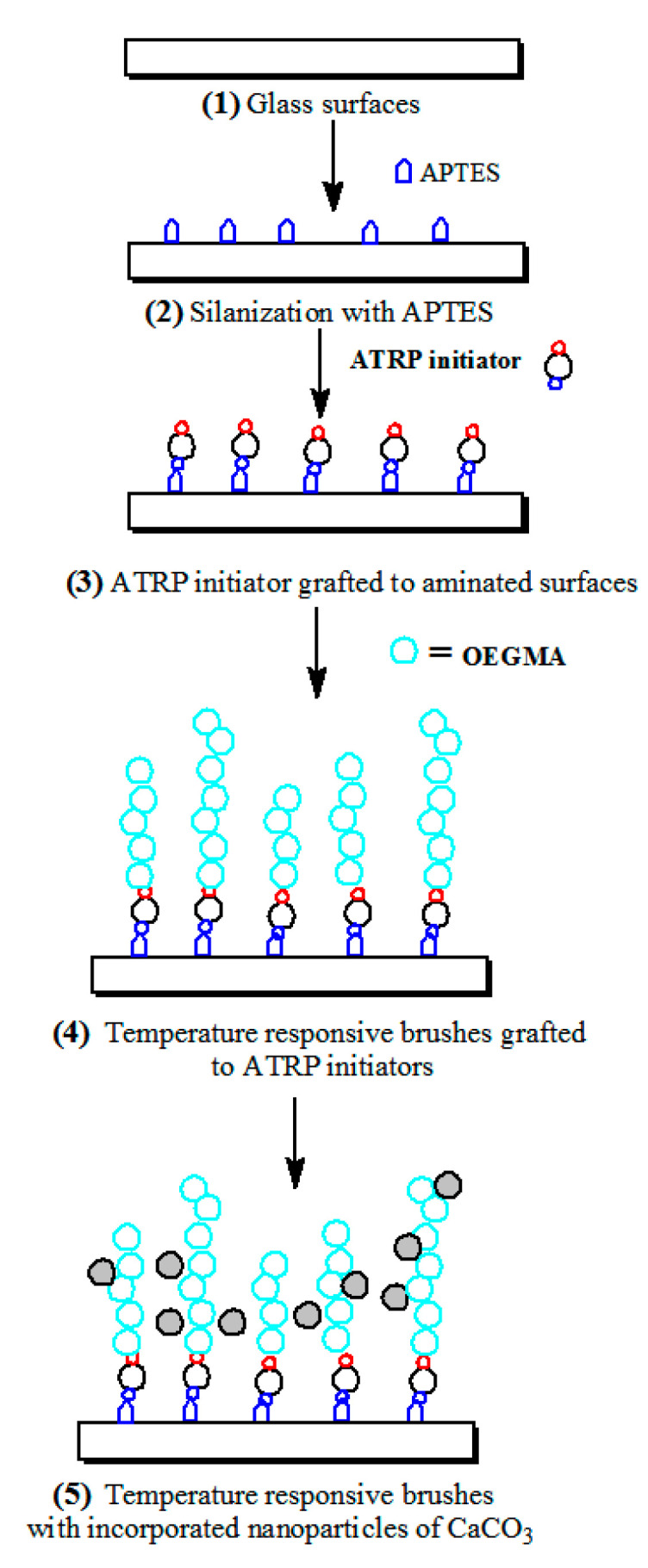
Functionalization of glass surface (**1**) with amino-terminated (3-aminopropyl)triethoxysilane (APTES) film (**2**); subsequent grafting of atom transfer radical polymerization (ATRP) initiator (**3**); polymerization of oligo(ethylene glycol) methyl ether methacrylate (OEGMA), initiated by ATRP initiator and resulting in poly(di(ethylene glycol)methyl ether methacrylate) (POEGMA) (**4**) brushes; followed by incorporation of CaCO_3_ nanoparticles (NPs) into grafted polymer brushes, resulting in the nanocomposite coatings (**5**).

The procedure of modification is sketched in Scheme 1. Glass plates with grafted ATRP were placed in test tubes, deoxygenated by nitrogen purging or vacuum/nitrogen cycling. Methanol (16 mL), water (4 mL) and di(ethylene glycol)methyl ether methacrylate)—OEGMA (34.8 g, 186.0 mmol) were mixed in a round-bottomed flask sealed with a septum, and deoxygenated by bubbling through nitrogen for 10–15min. Then, CuBr_2_ (7.4 mg, 0.033 mmol), 2,2′-dipyridyl (51.5 mg, 0.33 mmol) and sodium L-ascorbate (65.3 mg, 0.33 mmol) were added, and the headspace was purged with nitrogen. The mixture was stirred to dissolve the solids. Subsequently, the solution was syringed over the substrates in the deoxygenated tubes or simply poured over the substrates in a screw-top jar, which was then resealed. The samples were allowed to polymerize at ambient temperature. After 12 h of the polymerization, the samples were removed and washed with ethanol and water.

#### 2.2.3. Incorporation of CaCO_3_ NPs into Grafted Polymer Brushes

For mineralization of POEGMA-grafted brush coatings, 1M solution of CaCl_2_ and 1M solution of Na_2_CO_3_ were used. Before the mineralization was carried out, the samples with POEGMA-grafted brush coatings were preliminarily dipped in CaCl_2_ solution placed in a plastic container for 10 min. After that, the samples were dipped in Na_2_CO_3_ solution for 5 min, after which they were removed from the solution and set aside for completion of the crystallization process. Finally, the treated samples were washed with deionized water and dried in an oven at 40 °C for 20 min. Furthermore, the same procedure was repeated, and thus, two mineralization stages were performed.

### 2.3. Characterization of Coatings

#### 2.3.1. ToF-SIMS Analysis

ToF-SIMS spectra and images were collected using the TOF.SIMS 5 (ION-TOF GmbH, Munster, Germany) equipped with liquid metal ion gun, and Bi_3_^+^ ion clusters (30 keV) were used as the analysis beam (size of analyzed areas 200 µm × 200 µm). The apparatus was working in static mode conditions (an ion dose density lower than 10^12^ ion per cm^2^), and a low energy electron flood gun was used for charge compensation. Mass resolution (*m*/Δ*m*) at the C_4_H_5_ (*m*/*z* = 53) peak was greater than 8100 for all collected spectra.

#### 2.3.2. XPS Analysis

The X-ray photoelectron spectroscopy measurements were performed with a PHI VersaProbe II apparatus (Physical Electronics, MN, USA). The samples were irradiated with a focused monochromatic Al Kα (E = 1486.6 eV) X-ray beam with a diameter of 100 μm, and the beam was rastered over the area of 400 × 400 μm^2^. Analyzer’s pass energy was set to 46.95 eV, and double neutralization with electrons and low energy monoatomic Ar^+^ ions was used to avoid charging effects. Spectra were referenced to the neutral (C–C) carbon C 1 s peak, at a binding energy of 284.80 eV.

#### 2.3.3. Water Contact Angle Measurements (CA)

Static contact angle measurements were performed by means of the sessile drop technique using a KrussEasyDropDSA15 (KRÜSS GmbH, Hamburg, Germany) instrument. To determine the thermal response of the grafted brush coatings, the Peltier temperature-controlled chamber was used to carry out the measurements at temperatures ranging from 5 to 40 °C. The temperature was measured by a thermocouple in contact with the sample surface. Contact angle values were collected from 10 different spots and expressed as the average.

#### 2.3.4. Atomic Force Microscopy (AFM)

The commercially available Agilent 5500 system (Keysight, CA, USA) was used to examine the surface topography. The measurements were performed in air, using the non-contact mode with non-coated super sharp silicon probes.

#### 2.3.5. Ellipsometry

The thickness of the polymer brushes was measured by spectroscopic ellipsometer (SpectraRey/3, SENTECH Instruments GmbH, Berlin, Germany) equipped with a micro spot. The ellipsometric angles, Ψ and Δ, were determined for wavelengths λ in a spectral range between 320 and 800 nm. The measurements were taken at angles of incidence and detection of 56° and 70°. The polymer layers were modeled as a Cauchy layer of thickness d with a refractive index n(λ) of the form:

n(λ) = n_0_ + n_1_λ^−2^ + n_2_λ^−4^ (1)

Layer thickness d and the set of n_0_, n_1_, n_2_ parameters were varied numerically to achieve the best agreement between the model and the measured values, ∆(λ) and Ψ(λ). Refractive indices of the glass substrates were measured independently within the same spectral range and were accounted for in the model.

### 2.4. Cell Test

Cell culture: Three cell lines were used in this study, i.e., keratinocytes from histologically normal skin (HaCaT), WM35 cell line from the primary melanoma site of the patient’s skin diagnosed with radial growth phase (RGP) melanoma and osteoblastic cell line MC3T3-e1. HaCaT and MC3T3-e1 cell lines were purchased from ThermoFisher Scientific (Waltham, MA, USA) and WM35 cells were purchased from ATCC (Manassas, VA, USA).

HaCaT cells were cultured in the EMEM (Eagle’s minimum essential medium; LGC Standards, Teddington, United Kingdom) and WM35 cells were cultured in the RPMI-1640 (cell culture medium developed at Roswell Park Memorial Institute in 1966 by Moore and his co-workers, Sigma-Aldrich (Darmstadt, Germany)), both supplemented with a 10% heat-inactivated fetal bovine serum (FBS, Sigma-Aldrich, Darmstadt, Germany) and 1% antibiotics (Penicillin–Streptomycin–Neomycin Solution Stabilized, Sigma-Aldrich, Darmstadt, Germany) in culture flasks, in a CO_2_ incubator providing 95% air/5% CO_2_ atmosphere. Pre-osteoblastic MC3T3-E1 cells were cultured in MEM-alpha glutaMAX-1™ (Cat. No. 32561-029), ThermoFisher Scientific (Waltham, MA, USA) supplemented with 10% FBS, 2 mM glutamine, and 100 μg/mL penicillin/streptomycin. The media were replaced every 3 days, and the cells were maintained in a humidified incubator at 5% CO_2_ and 37 °C (Innova CO-170, New Brunswick Scientific, Enfield, CT, USA).

The POEGMA-grafted brush coatings with and without embedded CaCO_3_ nanoparticles were put into the Petri dish (35 mm in diameter) and sterilized for 3 min in 99.8% ethanol (POCH, Gliwice, Poland). After sterilization, a solution with of HaCaT or WM35 cells (80,000 cells per mL in the culture medium) was placed over the coatings. Next, the Petri dish was moved into the CO_2_ incubator for 1, 3 and 6 days. The experiments were repeated at least twice for each cell line and a timepoint.

Cell viability test: MC3T3-e1 cells were seeded into 6-well cell culture plates on the surface of the samples at a cell density of 10×10^4^ well and incubated 24, 72 and 144 h at 37 °C under 5% CO_2_. After that, cells were incubated (Innova CO-170, New Brunswick Scientific, Enfield, CT, USA) at 37 °C for 4 h; subsequently, 10 μL of fluorescence dye was added to each well (Alamar Blue, Sigma-Aldrich, Darmstadt, Germany). In the last step, fluorescent (540/610 nm) intensity was measured by a spectrophotometer (Synergy H1 Multi-Mode Reader, ThermoFisher Scientific, Waltham, MA, USA).

For WM35 and HaCaT cells, before fluorescence staining of actin filaments and the cell nucleus, cultured cells were pre-fixed to the substrate by adding a 1 mL solution of 3.7% of paraformaldehyde (Fluka, Charlotte, NC, USA) to the culture medium for 2 min at 37 °C. Then, cells were washed with phosphate-buffered saline (PBS, Sigma-Aldrich, Darmstadt, Germany) 3 times for 2 min. Afterwards, the sample was immersed in the solution of 3.7% of paraformaldehyde (Fluka, Charlotte, NC, USA) for 20 min at room temperature to fix the cells firmly. After fixation, samples with cells were rinsed twice with the PBS buffer for 2 min. After that, a cold solution (4 °C) of 0.2% Triton X-100 (Sigma-Aldrich, Darmstadt, Germany) was added for 4 min, followed by washing samples with the PBS buffer for 2 min. Next, the cells were incubated with phalloidin conjugated with a 1:200 solution Alexa Fluor 488 dye (Invitrogen, Carlsbad, CA, USA) for 40 min, and subsequently, cell nuclei were incubated with a 1:5000 solution containing Hoechst dye (Sigma) for 14 min. The fluorescent images were collected from at least three repetitions carried out for each cell line and a timepoint. For each experimental run, at least 10 fluorescent images from two or three cover slips with stained cells were collected. Proliferation index was expressed as the ratio between the number of the cells on the surface after a given time of cultivation and the number of the cells on the surface after 24 h culture.

Fluorescence imaging: WM35 and HaCaT cell growth on the examined substrates was traced using a fluorescence Olympus IX51 microscope equipped with a 100 W Mercury light source (U-LH100HG, Olympus, Tokyo, Japan), U-MWIG2 filter (λexit = 530–550 nm, λemit = 590 nm, Olympus, Tokyo, Japan)) and U-MNB2 one (λexit = 470–490 nm, λemit = 520 nm, Olympus, Tokyo, Japan)). To study the growth of fibroblasts, the first filter was used to record images of actin filaments while the later one to detect fluorescently labeled cell nuclei.

Fluorescent images were recorded using the XC30 digital camera (Olympus, Tokyo, Japan)). The maximum resolution of images captured by this camera is 2080 × 1544 pixels. All images were recorded using CellSense Dimensions (Olympus) software with a 20x (Universal Plan Fluorite) lens. To prove the reproducibility of the results, the experiments were repeated at least three times for each cell line for each timepoint. For each experimental sequence, three identical samples were prepared and measured.

To visualize the viable MC3T3-e1 cells, a Nikon TI (Nikon Instruments Inc., Melville, NY, USA) fluorescence microscope with the 10X objective was used. After 24, 72 and 144 h of incubation on samples, cell layers were then stained with Calcein AM. Cells were incubated with a medium containing 0.1 mM of the reagent for 10 min at RT.

### 2.5. Statistical Analysis

All statistical analysis was performed by multivariate ANOVA, using Origin (OriginLab, Northampton, MA, USA). Statistical significance between groups was determined by performing Bonferroni’s post-hoc analysis. Statistical significance was achieved for *p* < 0.05.

## 3. Results and Discussion

In the present work, the temperature-responsive POEGMA-grafted polymer brush coatings with low concentration of the embedded CaCO_3_ NPs were fabricated (Scheme 1). The composition, thickness, morphology and wettability of the resulting coatings were analyzed using ToF-SIMS, ellipsometry, SEM, AFM, XPS and CA measurements, respectively, and are described in detail in Section 3.1. In turn, the impact of the embedded CaCO_3_ NPs on adhesion and growth of three cell lines of different types (normal skin (HaCaT), cancer cells from the primary melanoma site (WM35), and osteoblastic cell line (MC3T3-e1)) is presented in Section 3.2.

### 3.1. Fabrication and Characterization of the POEGMA-Grafted Brush Coatings with Embedded CaCO_3_ NPs (ToF-SIMS, XPS, Ellipsometry, CA, SEM, AFM)

The fabrication process of the poly(di(ethylene glycol)methyl ether methacrylate)POEGMA-grafted brush coatings with embedded CaCO_3_ NPs is depicted in Scheme 1.

Grafted temperature-responsive polymer brushes attached to the glass surface were prepared in a three-step process using ATRP polymerization. For this purpose, the glass surface was functionalized firstly by (3-aminopropyl)triethoxysilane (APTES) and then by ATRP molecules. The thicknesses of the grafted brush coatings can be easily tuned by the time of the ATRP polymerization of oligo(ethylene glycol) methyl ether methacrylate (OEGMA). Subsequently, uniform CaCO_3_ NPs embedded in POEGMA-grafted brush coatings were successfully synthesized using mineral precipitation from solutions of CaCl_2_ and Na_2_CO_3_. In previous papers [52,53,54,55,56,57,58], the synthesis of CaCO_3_ NPs in the presence of poly(ethylene oxide)-*block*-poly((meth)acrylic acid), poly(ethylene oxide)-*block*-polyethylenimine and poly(ethylene glycol)-*block*-poly-(methacrylate-*graft*-poly(acrylic acid)) have been reported. In recent work [59], novel composite scaffolds based on polymeric polycaprolactone fibers coated with porous calcium carbonate structures were easily fabricated using a mineralization procedure of treatment in a CaCl_2_/Na_2_CO_3_ reaction mixture, similar to our methodology but for different application—amplification of weak Raman signals from molecules.

The thickness and refractive index of the coatings were analyzed using ellipsometry. The typical thickness of the grafted APTES, measured by ellipsometry for the conditions used for glass functionalization, was equal to 0.5 nm. The thickness of the ATRP films did not exceed 0.8 nm. The thicknesses of POEGMA-grafted brush coatings and POEGMA with CaCO_3_ NPs in a dry state were 110.6 ± 9.3 nm and 117.4 ± 14.1 nm, respectively. In turn, the refractive index of the POEGMA coatings was 1.52 ± 0.02, whereas for POEGMA films with CaCO_3_ NPs, it equals 1.61 ± 0.05. These results imply that the incorporation of CaCO_3_ NPs into the POEGMA polymer matrix has a very weak impact on the thickness of the coatings, contrary to their optical properties, which change significantly.

**Figure 1:**
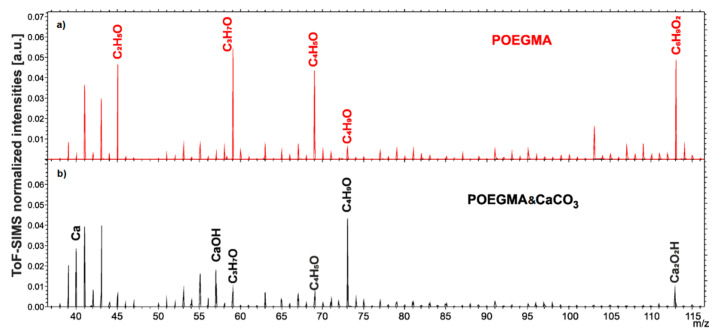
Representative positive ion time of flight secondary ion mass spectroscopy (ToF-SIMS) spectra of the POEGMA-grafted brush coatings (**a**) with embedded CaCO_3_ NPs (**b**). The signals characteristic and unique for the coating following each fabrication step are marked with secondary ion names, respectively.

To prove the presence of CaCO_3_ NPs in the polymer brushes, the chemical composition of formed coatings was analyzed using the ToF-SIMS technique. The ToF-SIMS spectra depict the series of fragments characteristic of polymer and inorganic particles. The normalized intensities of the ToF-SIMS peaks are shown in Figure 1. Presented spectra clearly depict a series of peaks C_2_H_5_O^+^, C_3_H_7_O^+^, C_4_H_5_O^+^ and C_6_H_9_O_2_^+^ characteristic for POEGMA [44,60,61]. In addition, intensities of these peaks decrease after the incorporation of CaCO_3_ NPs and new signals characteristic of those NPs are observed (Ca^+^, CaOH^+^, Ca_2_O_2_H^+^). Those observations confirm the effectiveness of the proposed process of fabrication of polymer brushes with embedded CaCO_3_ NPs.

**Figure 2:**
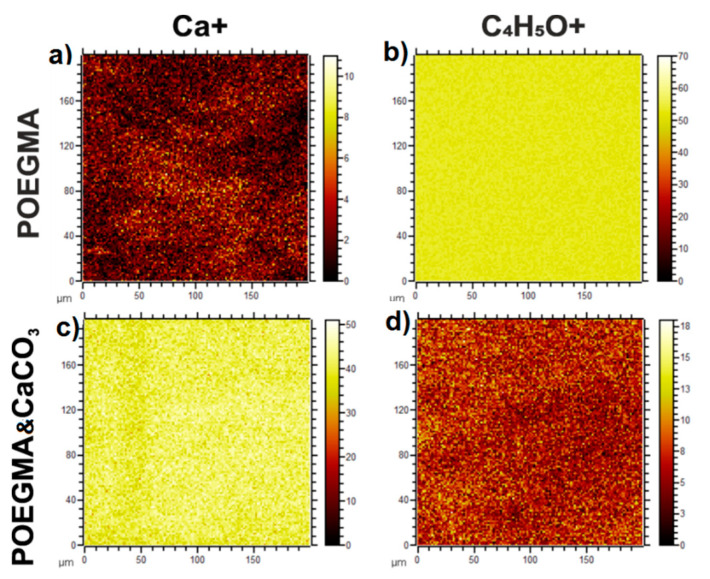
ToF-SIMS chemical imaging of the POEGMA-grafted brush coatings before (**a**,**b**) and after (**c**,**d**) mineralization.

The homogeneity and chemical composition of the samples before and after mineralization were confirmed additionally using ToF-SIMS images collected during analysis. The ToF-SIMS images were recorded for C_4_H_5_O^+^ (Figure 2b,d) and Ca^+^ (Figure 2a,c) ions, corresponding to POEGMA and CaCO_3_ NPs, respectively [60]. For both polymer coatings, a homogeneous distribution of C_4_H_5_O^+^ ions (Figure 2b,d) typical for POEGMA is visible. Moreover, for the films without CaCO_3_ NPs (Figure 2a), a not-so-significant amount of Ca^+^ ions (counts < 10) is detected. In contrast, for polymer coatings with embedded nanoparticles, a strong (counts > 50), uniform signal corresponding to Ca^+^ ions (Figure 2c), and characteristic for nanoparticles, is recorded.

**Figure 3:**
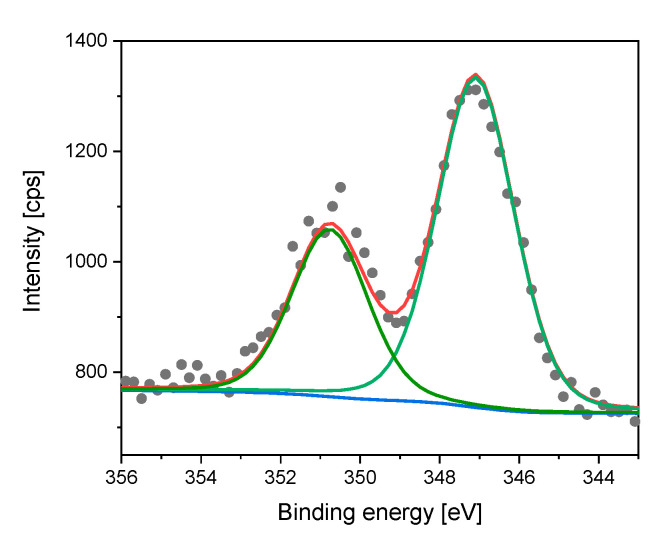
Ca 2p XPS spectra of POEGMA-grafted brush coating with embedded CaCO_3_ (Ca 2p 3/2 line position 347.2 eV, multiplet splitting 3.67 eV).

Results of the XPS analysis are presented in Figure 3 and Table 1. Carbon C1s spectra were fitted with three peeks corresponding to C–C (284.8 eV) C–O or C–N (286.5 eV) and O–C=O (288.5 eV) bonds. The peak positions are only slightly different from those determined for “pure” POEGMA and presented in our earlier work [62]. In addition, the relative intensities of the peaks vary slightly from those previously measured. On this basis, it can be concluded that the procedure of introducing CaCO_3_ NPs into POEGMA has no significant effect on its chemical state. It should also be stated that this procedure is effective as indicated by the Ca concentration of 1.88 ± 0.53% evaluated from XPS analysis for the POEGMA coatings with CaCO_3_. In addition, the position of the Ca 2p 3/2 peak, determined for the value of 347.2 eV, should be related to the CaCO_3_ compound [63]. Results of the XPS analysis allow us to determine conditional designation for coatings defined as coatings with “low” concentration of CaCO_3_ NPs. The required “low” concentration is very important, as for “high’’ concentrations of the NPs, the temperature-responsive properties of the coatings disappear [49].

**Figure 4:**
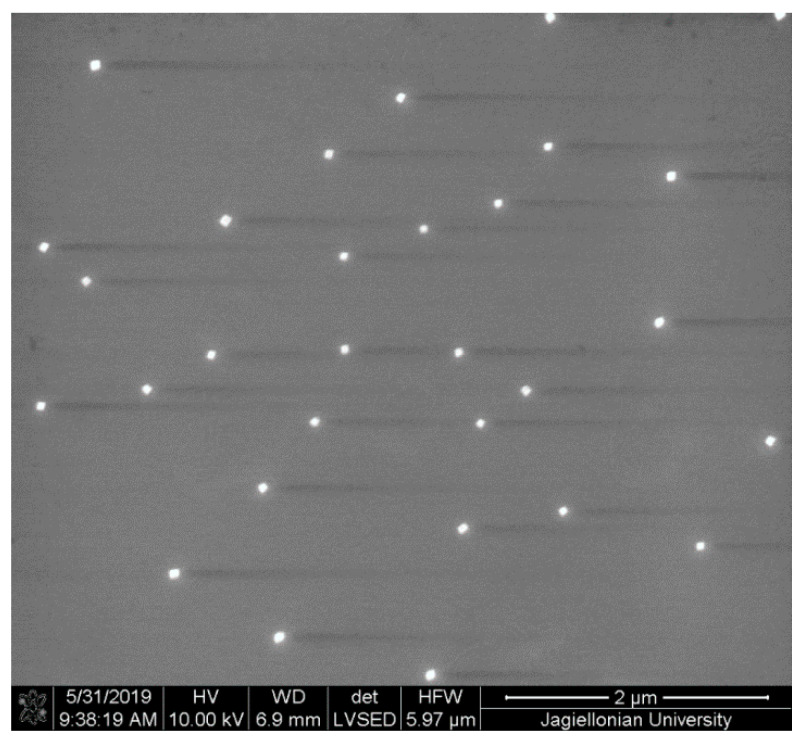
Representative SEM image of CaCO_3_ NPs incorporated in POEGMA-grafted brush coatings.

The SEM images (Figure 4) recorded for samples with CaCO_3_ NPs incorporated in POEGMA-grafted brush coatings demonstrated uniformly distributed CaCO_3_ NPs possessing the square shape. Their sizes did not exceed several tens of nanometers. It is well known [52,64,65,66] that CaCO_3_ NPs during synthesis form a mixture of vaterite (spherical porous) and calcite (cubic crystals) forms, and the ratio between these forms depends on the conditions of the synthesis. In addition, the vaterite usually transforms to a calcite, which is a more stable CaCO_3_ polymorphic form, representing cubic crystals, as depicted in Figure 4.

**Figure 5:**
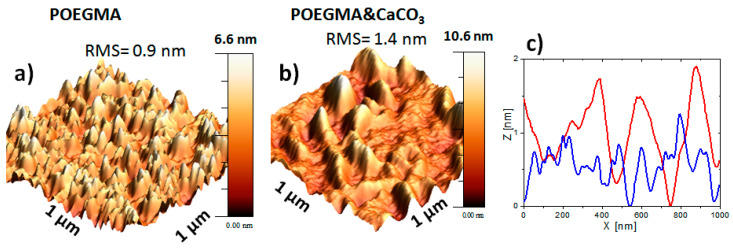
Images of the POEGMA-grafted brush (**a**) and POEGMA-grafted brushes with incorporated CaCO_3_ NPs (**b**) recorded using AFM working in topography (**a**,**b**) mode. Cross-line analysis corresponds to the topography (**c**) of the samples without (blue) and with CaCO_3_ NPs (red).

Images the POEGMA-grafted brushes and POEGMA-grafted brushes with incorporated CaCO_3_ NPs recorded using AFM, working in topography mode, are presented in Figure 5. Incorporation of the CaCO_3_ strongly transforms the structure of the coating fromrelatively smooth (Figure 5a,c, blue line) to more structured with good expressed large elevations (Figure 5b,c, red line).

**Figure 6:**
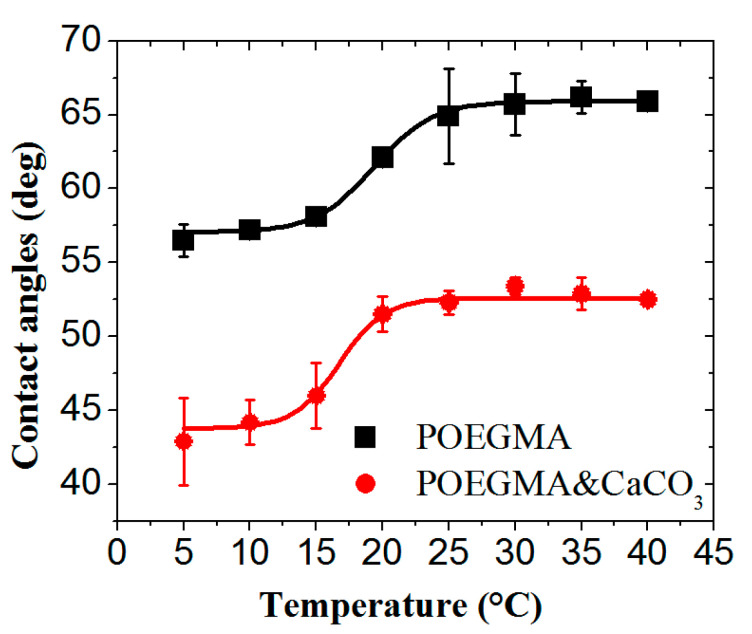
Temperature dependence of the water contact angle determined for the POEGMA-grafted brush coatings and the POEGMA-grafted brush coatings with embedded CaCO_3_ NPs.

To examine the temperature-sensitive properties of the POEGMA-grafted brush coatings with and without embedded CaCO_3_ NPs, contact angles of sessile water droplets were measured between 5 and 40 °C. The results, presented in Figure 6, show a thermal response of the POEGMA-grafted brush coatings, with the water contact angle changing from 56 to 65 deg. The wettability changes also for the POEGMA-grafted brush coatings with embedded CaCO_3_ NPs (Figure 6, red circles). In this case, the increasing temperature induces changes in the water contact angle of nearly 10 deg, from 42 deg to 52 deg. The thermal response of the wettability is manifested by a well-defined transition at 16–18 °C for both coatings. Similar to the results described for incorporated silver nanoparticles [67], the curves of the temperature dependences of water contact angles determined for the POEGMA-grafted brush coatings and the POEGMA-grafted brush coatings with embedded CaCO_3_ NPs have a similar character but different values of the contact angles. More hydrophilic character of the surface for POEGMA-grafted brush coatings with embedded CaCO_3_ NPs may be related to a higher surface energy of CaCO_3_.

Presented results of physicochemical analysis of the coatings suggest a successful fabrication of the temperature-responsive POEGMA-grafted polymer brush coatings with a low concentration of the embedded CaCO_3_ NPs. The size of nanoparticles, determined using SEM, does not exceed several tens of nanometers, and the concentration of the calcium in polymer coatings, provided by XPS measurements, is about 2%.

### 3.2. Impact of the POEGMA Coatings with a Low Concentration of Embedded CaCO_3_ Nanoparticles on Behavior of the Cellular Lines

The presence of biomolecules or polymers on the surface of the NPs has a significant impact on their absorption, bioaccumulation, and biotransformation and can lead to unforeseen changes in the toxicity [16,68]. Thus, differences in the bioavailability of gold NPs were noted previously [69], depending on whether they were uncoated or coated with polyethylene glycol. In turn, a completely novel approach is based on incorporation of the NPs in “smart”-grafted coatings and switching their biological activity using external stimulus [70,71,72,73,74]. In our previous work [49], we demonstrated the impact of the POEGMA with a low concentration of the silver nanoparticles on cytotoxicity of POEGMA-based nanocomposite coatings. The comparison of the growth of HaCaT cells on POEGMA-grafted brush coatings and on POEGMA coatings with AgNPs suggested that cells grow faster on polymer brushes with nanoparticles. At the same time, for WM35 cancerous cells, rather an opposite effect was noticed. These results indicate that AgNPs embedded in polymer brush do not have any significant cytotoxic effects towards normal cells and suggest a slight anti-cancer impact of AgNPs. It was noted in several literature sources [37,42,75,76] that cells demonstrate a low adhesion on POEGMA-grafted brush coatings. On the other hand, CaCO_3_ NPs or calcite monolith are highly attractive for cell growth [77]. In addition to the ideas proposed before [59,78], to incorporate inorganic nanoparticles polymer microparticles, in this study, we designed the temperature-responsive POEGMA brush coatings with a “low” concentration of the embedded CaCO_3_ NPs as biologically active substrate to test their impact on different types of the cells. In work presented by A. Kamba et al. [79], very low toxicity of the CaCO_3_ NPs cultured with cell cultures has been noted. In contrast, in studies performed by M. Zhang et al. [80], a cytotoxic effect of CaCO_3_ NPs to breast cancer cell line MDA-MB-231 was noted, which was manifested by a change in the size and the morphology of cells, the formation of large cytoplasmic vacuoles, the inhibition of proliferation, and the induction of apoptosis.

**Figure 7:**
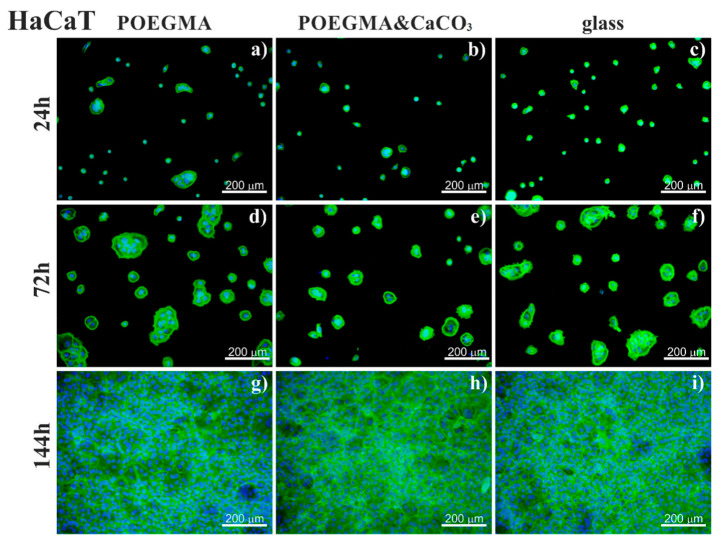
Fluorescence images of HaCaT cells after 24 (**a**–**c**), 72 (**d**–**f**) and 144 h (**g**–**i**) of cultivation on glass surface (**c,f**,**i**), POEGMA (**a**,**d**,**g**) and POEGMA-grafted brush coatings with incorporated CaCO_3_ NPs (**b**,**e**,**h**).

To verify impact of the fabricated nanocoatings with low concentration of CaCO_3_ NPs on cellular behavior, three different types of cell lines (normal skin (HaCaT), cancerous from the primary melanoma site (WM35) and osteoblastic cell line (MC3T3-e1)) were chosen. Representative fluorescence images recorded for 24, 72 and 144 h culture time are presented in Figures 7, 9 and 11, for HaCaT, WM35 and MC3T3-e1 cells, respectively. The amount of cells after 24 h of the cultivation demonstrates their ability of adhesion to the surface. In turn, their amount after 72 and 144 h of incubation indicates proliferative activity (proliferation index), which may be expressed as the ratio between the number of the cells on the surface after a given time of cultivation and the number of the cells on the surface after 24 h culture. For such definition of the proliferation index, it always equals 1 for 24 h of cultivation. In addition, viability of cells at each time point of culture is defined as the ratio between the number of cells on the examined substrate and that on the control sample.

**Figure 8:**
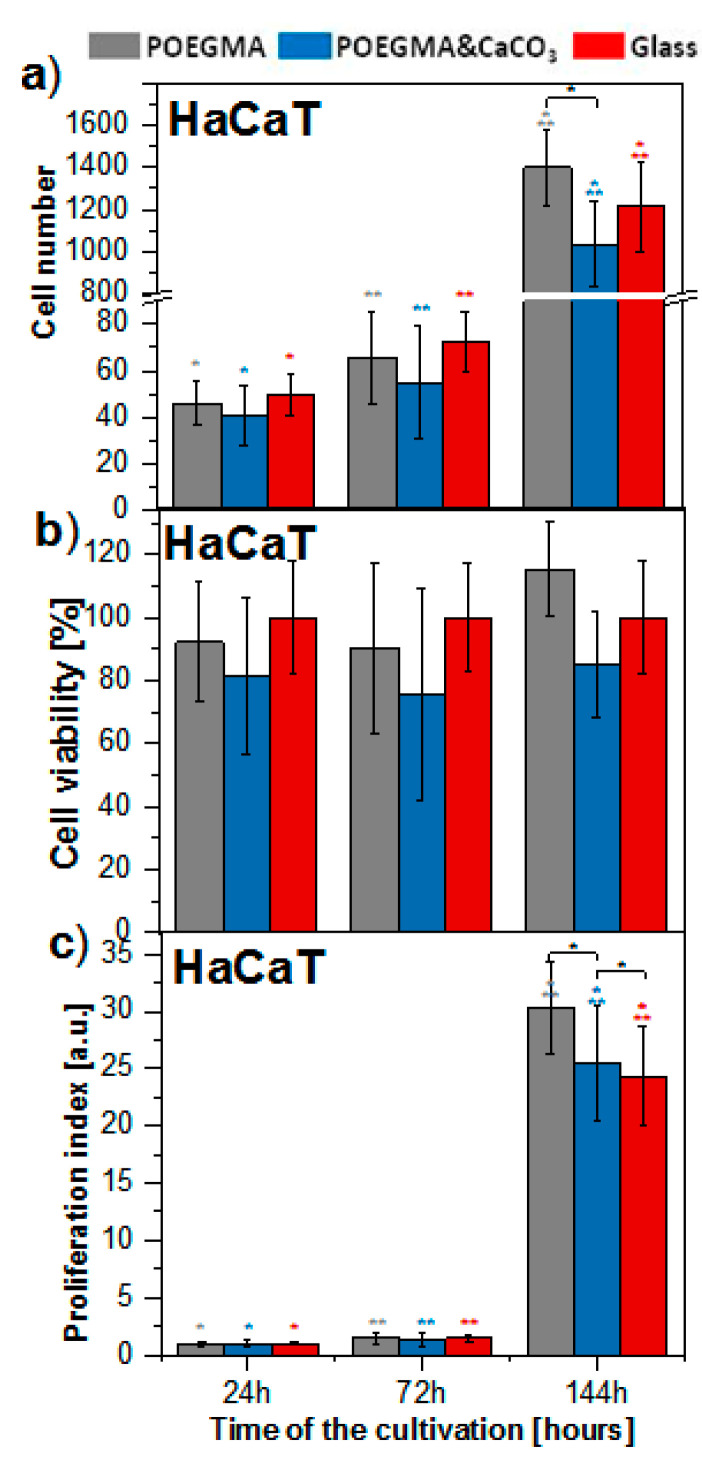
Impact of glass surface, POEGMA and POEGMA-grafted brush coatings with incorporated CaCO_3_ NPs on the amount (**a**), viability (**b**) and proliferation index (**c**) of HaCaT cells (*, ** significantly different from each other, *p* < 0.05). Data are presented as a mean ± standard deviation (SD) from *n* = 10 repetitions.

Surprisingly, adhesion of the HaCaT cells on control surface and potentially non-adhesive POEGMA coatings demonstrates similar tendency (results presented in Figure 7 and Figure 8a, 24 h of the cultivation) and it is slightly lower on the POEGMA with CaCO_3_ NPs (Figure 7b and Figure 8a, 24 h). Cell viability, calculated for all culture times and presented in Figure 8b, is slightly reduced for cells cultivated on the POEGMA with CaCO_3_ NPs (Figure 8b) as compared to glass surfaces. However, it does not decrease below 80%; therefore, according to the ISO 10993-5 standard, it can be considered as non-cytotoxic [81]. For HaCaT cells cultured on the POEGMA coatings, a similar tendency is observed for 24 and 72 h, whereas for 144 h improved cell viability in comparison to the control is recorded. Calculated proliferation index (Figure 8c) suggests an approximately equal growth of the HaCaT cells on all surfaces for 72 h of the incubation (Figure 7 and Figure 8c, 72 h) and slightly increased POEGMA coatings for 144 h (Figure 7 and Figure 8c, 144 h).

**Figure 9:**
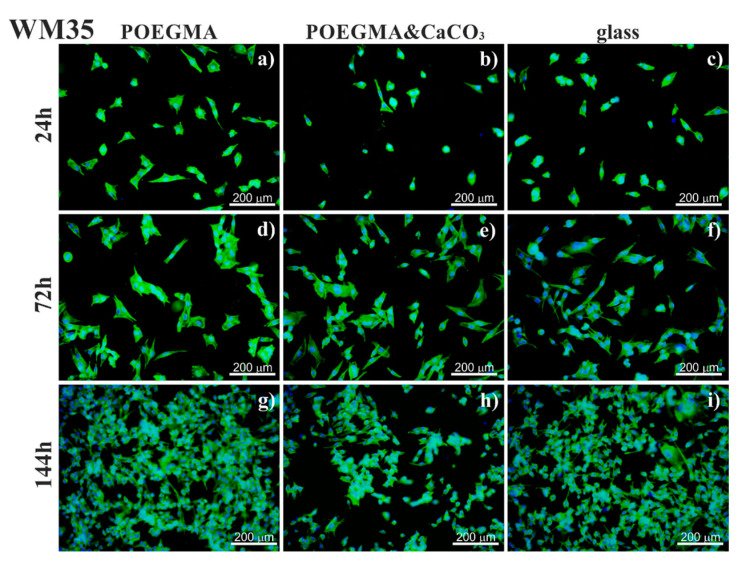
Fluorescence images of cancerous WM35 cells after 24 (**a**–**c**), 72 (**d**–**f**) and 144 h (**g**–**i**) of cultivation on glass surface (**c**,**f**,**i**), POEGMA (**a**,**d**,**g**) and POEGMA-grafted brush coatings with incorporated CaCO_3_ NPs (**b**,**e**,**h**).

**Figure 10:**
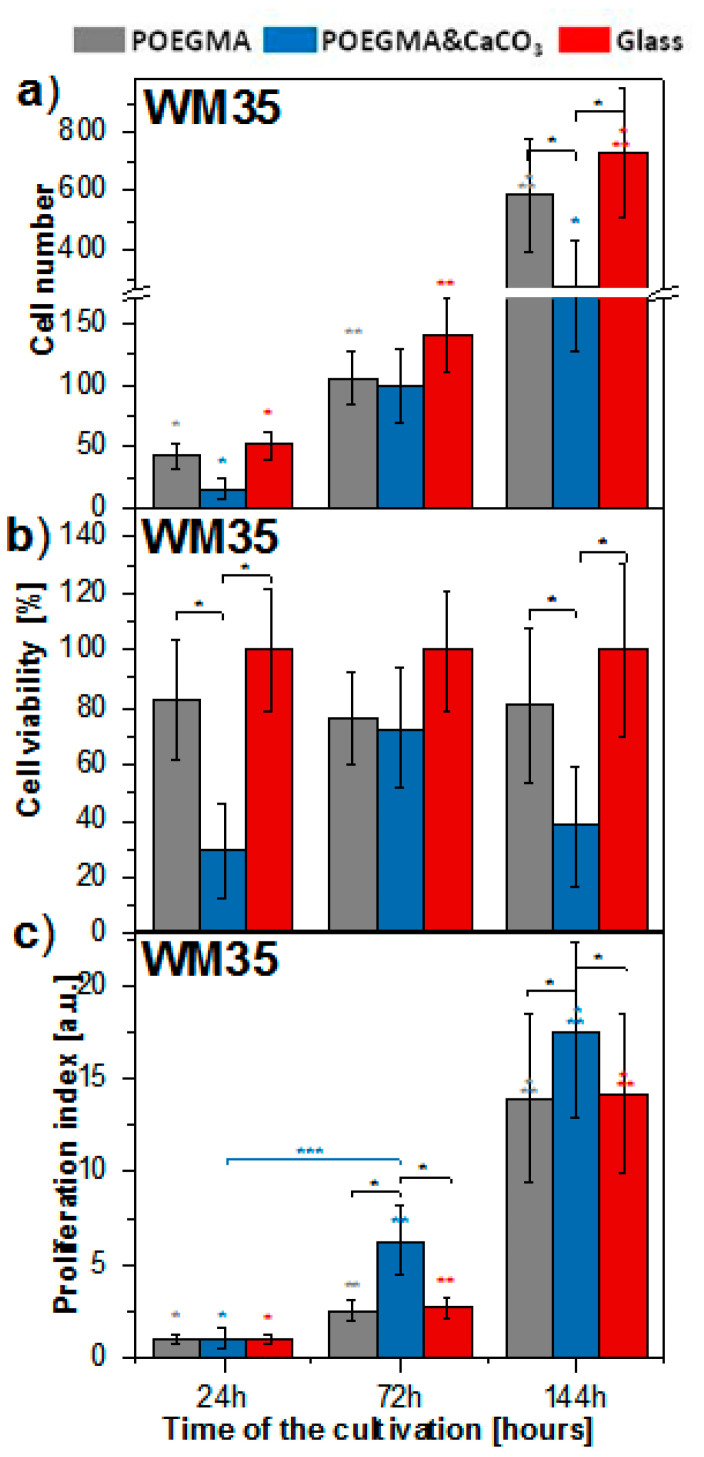
Impact of glass surface, POEGMA and POEGMA-grafted brush coatings with incorporated CaCO_3_ NPs on the amount (**a**), viability (**b**) and proliferation index (**c**) of WM35 cells (*, **, *** significantly different from each other, *p* < 0.05). Data are presented as a mean ± standard deviation (SD) from *n* = 10 repetitions.

In contrast, for WM35 cancerous (melanoma) cells on the POEGMA coating with embedded CaCO_3_ NPs, the analysis of fluorescence micrographs depicts a very low adhesion (Figure 9 and Figure 10a, 24 h), whereas the adhesion of WM35 cells on the control glass sample and POEGMA coatings without CaCO_3_ NPs were almost identical. Cell viability calculated after 24 h of the cultivation for the WM35 cells is very low (≈ 30%) on POEGMA coatings with CaCO_3_ NPs and slightly reduced (≈ 80%) on POEGMA coatings in comparison to glass surface (Figure 10b). The viability of the WM35 cells on POEGMA coatings for longer culture times was similar as for 24 h of the cultivation. In contrast to the POEGMA coatings, the viability of the WM35 cells on POEGMA coatings with CaCO_3_ NPs increases up to 70% for 72 h of the cultivation and decreases to 30% for 144h.

Contrary to the adhesive behavior of WM35 cells, the proliferation index was significantly higher for POEGMA coating with embedded CaCO_3_ NPs as compared to both control as well as “pure” POEGMA coatings (Figure 9 and Figure 10a, 72 and 144 h).

The comparison of the cell viability and proliferation index calculated for WM35 cells cultured on POEGMA coatings with embedded CaCO_3_ NPs suggests that the reduced number of cells, as compared with the control sample, is related rather with the poor adhesive properties of the coating than with its cytotoxicity.

**Figure 11:**
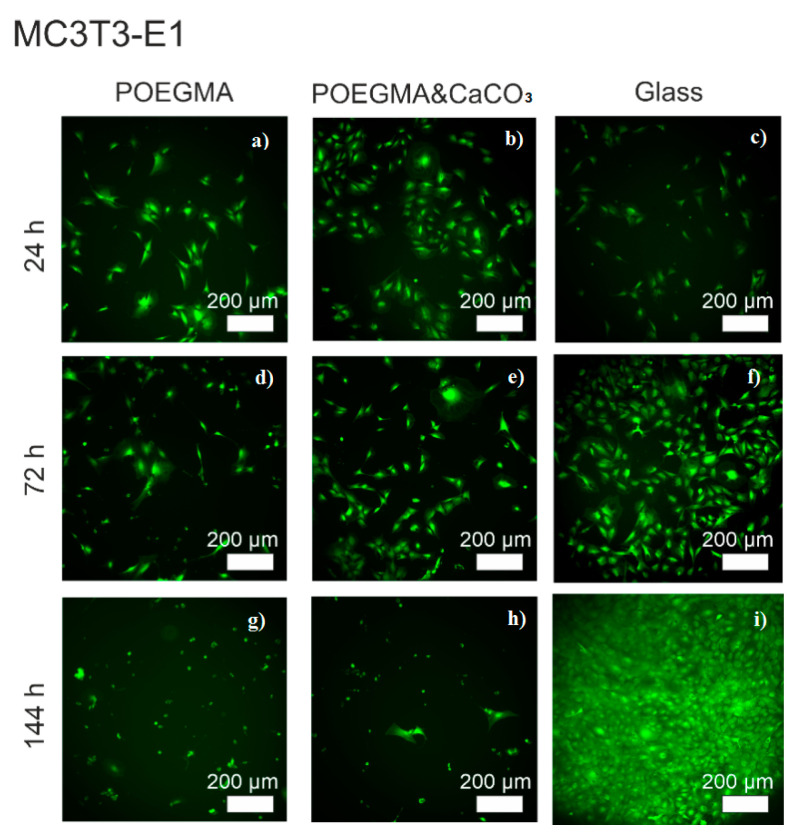
Fluorescence images of osteoblastic cell line MC3T3-e1 (Calcein AM) after 24 (**a**–**c**), 72 (**d**–**f**) and 144 h (**g**–**i**) of cultivation on glass surface (**c**,**f**,**i**), POEGMA (**a**,**d**,**g**) and POEGMA-grafted brush coatings with incorporated CaCO_3_ NPs (**b**,**e**,**h**).

**Figure 12:**
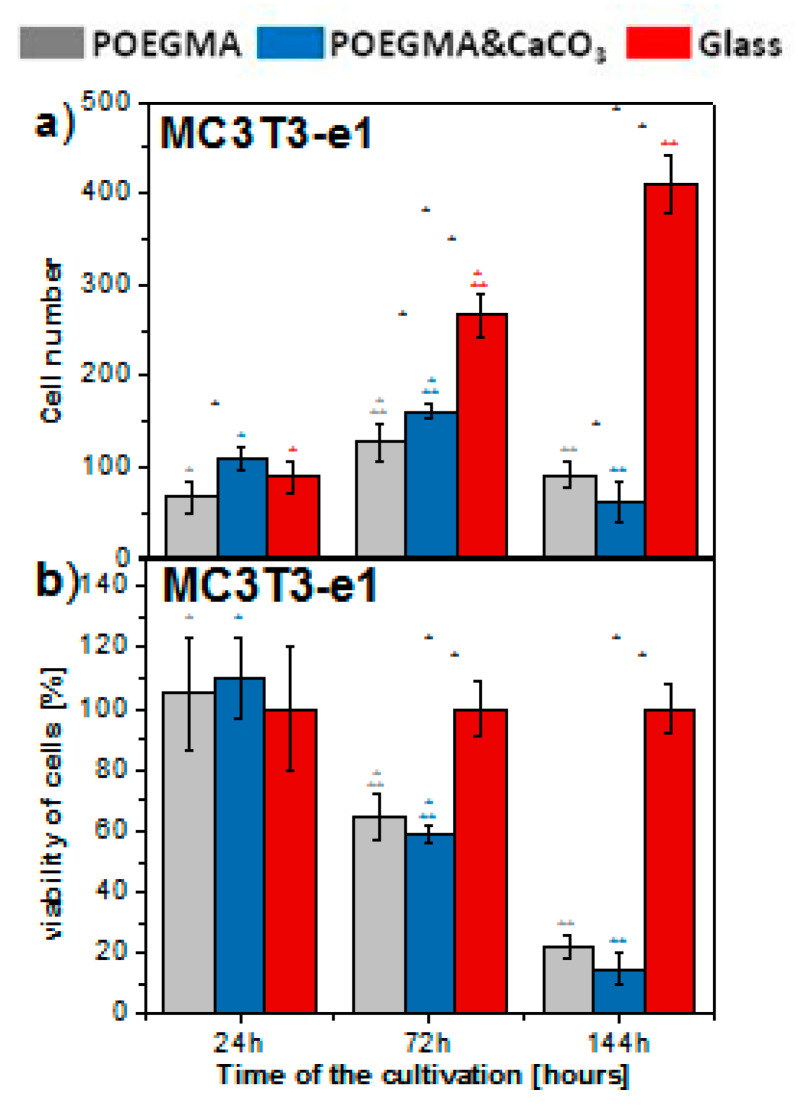
Impact of glass surface, POEGMA and POEGMA-grafted brush coatings with incorporated CaCO_3_ NPs on the amount (**a**) and viability (**b**) of osteoblastic cell line MC3T3-e1 (*, ** significantly different from each other, *p* < 0.05). Data are presented as a mean ± standard deviation (SD) from *n* = 10 repetitions.

A more complicated situation was observed for the osteoblastic cells (Figure 11 and Figure 12), where the completely anti-adhesion effect was expected, based on the previous studies for osteoblastic cell line MC3T3 on POEGMA coatings [75,76]. In contrast to the data reported in the literature, adhesion of the osteoblasts was observed for the examined coatings after 1 day of cultivation. The chemical nature of the grafted brush coating as well as molecular mass and grafting density are important for adhesion and growth of the cells. As the information about these parameters is missing in references [75,76], the comparison between the coating properties and their potential impact on cellular behavior is not possible. The number of cells cultured on the POEGMA and POEGMA-grafted brush coatings with incorporated CaCO_3_ NPs was comparable with their number on the control sample. However, for longer culture times, cells start to detach from both POEGMA coatings, and their number is significantly reduced. This effect is visible also in cell viability (Figure 11 b), which significantly decreases for longer culture times. This phenomenon suggests a relatively low suitability of POEGMA coatings for cultivation of the osteoblastic cells.

In general, CaCO_3_ NPs demonstrate a very low cytotoxicity [16,79], and even improved cell growth on coatings containing CaCO_3_ NPs was described [82]. However, for some cell lines cultured in the presence of CaCO_3_ NPs, an endocytosis, the production of intracellular reactive oxygen species, membrane damage, and cell apoptosis were shown [16,83,84].

## 4. Summary and Conclusions

In the present work, we have successfully prepared and characterized novel nanocomposite materials based on temperature-responsive grafted brush coatings of POEGMA with low concentration of the embedded CaCO_3_ NPs. CaCO_3_ NPs are prospective material that can be used for the delivery of drugs, showing even a more pronounced biological activity than the use of drugs alone [85]. Grafted temperature-responsive polymer brushes attached to the glass surface were prepared in a three-step process using ATRP polymerization. Subsequently, uniform CaCO_3_ NPs embedded in POEGMA-grafted brush coatings were synthesized using mineral precipitation from solutions of CaCl_2_ and Na_2_CO_3_.

The average thicknesses of POEGMA-grafted brush coatings and POEGMA with CaCO_3_ NPs in a dry state were 110.6 ± 9.3 nm and 117.4 ± 14.1 nm, respectively. In turn, the refractive index of the POEGMA coatings was 1.52 ± 0.02, whereas for POEGMA films with CaCO3 NPs, it equals 1.61 ± 0.05. The presence of CaCO_3_ NPs in the polymer brushes, their shape and concentration, as well as chemical composition and morphology of formed coatings, were analyzed using ToF-SIMS, SEM, AFM and XPS techniques. The uniformly distributed CaCO_3_ NPs possessing the square shape with sizes not exceeding several tens of nanometers suggest polymorphic forms of calcite. The concentration of calcium in polymer coatings provided by XPS measurements is equal to approximately 2%, which corresponds to a low concentration of CaCO_3_ NPs embedded in POEGMA brush coatings. For this concentration of CaCO_3_ NPs, the temperature-responsive properties of the POEGMA coatings were retained after incorporation of NPs.

An impact of the low concentration of the embedded CaCO_3_ nanoparticles on cell adhesion and growth was studied in detail for three cell lines: keratinocytes from histologically normal skin (HaCaT), WM35 cell line from the primary melanoma site of the patient’s skin diagnosed with radial growth phase (RGP) melanoma and osteoblastic cell line (MC3T3-e1). These cells were cultured on glass modified with grafted POEGMA brush coatings and POEGMA brush coatings with the embedded CaCO_3_ nanoparticles. It can be postulatedthat the impact of POEGMA and POEGMA-grafted brush coatings with low concentration of the embedded CaCO_3_ NPs strongly depends on the type of the cell lines. In contrast to the non-adhesive properties of POEGMA-based coatings reported before [43,75], we did not find significant differences in cell adhesion for 24 h of the incubation on control (glass) surface and POEGMA coatings for all studied cell lines. The comparison between the number of cells cultured 24h on POEGMA-grafted brush coatings with incorporated CaCO_3_ NPs and on “pure” POEGMA coatings shows essential and slight reduction in adhesion for WM35 and HaCaT cell lines, respectively. At this time, the completely anti-adhesion effect described before for the osteoblastic cell line MC3T3-el on POEGMA coatings [75,76] was absent here and has been surpassed by the incorporation of nanoparticles. However, for longer culture times, the amount of cells for both POEGMA coatings (i.e., “pure” and with embedded NPs) was reduced by almost five times.

Future applications of the presented system require the tuning of the LCST towards the higher temperatures, which may be easily achieved by fabrication of the grafted copolymer brush coatings with properly adjusted composition. In work of [86], it was shown that copolymers synthesized on a base of di(ethylene glycol) methyl ether methacrylate with a low content of oligo(ethylene glycol) methyl ether methacrylate (≈10%) demonstrated an essential increase in the LCST. Similar results were presented in the work of [87], where the composition of temperature-responsive copolymer brushes based on oligo(ethylene glycol) methacrylates was tuned to obtain a collapse temperature of ∼35 °C. Based on the temperature-responsive properties of grafted brush coatings and CaCO_3_ NPs as a material with a controlled release of the biologicallyactive substances, we believe that future research will allow the development of new platforms for tissue engineering with improved growth of cells due to both the presence of nanoparticles and release of the biologically active substance from CaCO_3_ NPs.

## Figures and Tables

**Table 1:** Relative Intensities of C–C, C–O (C–N), and O–C=O signals.

Coating	Type of the Bond	BE (eV)	INT (%)
**POEGMA with CaCO_3_**	C–C	284.8	71
	C–O (C–N)	286.5	19
	O–C=O (C=O)	288.5	10
**POEGMA [52]**	C–C	284.8	72
	C–O (C–N)	286.4	21
	O–C=O (C=O)	288.4	7

## Data Availability

The data presented in this study are available on request from thecorresponding author.
